# Draft Genome Sequence of the Xylella fastidiosa CoDiRO Strain

**DOI:** 10.1128/genomeA.01538-14

**Published:** 2015-02-12

**Authors:** Annalisa Giampetruzzi, Michela Chiumenti, Maria Saponari, Giacinto Donvito, Alessandro Italiano, Giuliana Loconsole, Donato Boscia, Corrado Cariddi, Giovanni Paolo Martelli, Pasquale Saldarelli

**Affiliations:** a Institute for Sustainable Plant Protection, National Research Council (CNR), Bari, Italy; b Department of Physics, University of Bari Aldo Moro, Bari, Italy; c Department of Soil, Plant and Food Sciences, University of Bari Aldo Moro, Bari, Italy

## Abstract

We determined the draft genome sequence of the Xylella fastidiosa CoDiRO strain, which has been isolated from olive plants in southern Italy (Apulia). It is associated with olive quick decline syndrome (OQDS) and characterized by extensive scorching and desiccation of leaves and twigs.

## GENOME ANNOUNCEMENT

Xylella fastidiosa is a xylem-restricted Gram-negative bacterium and the agent of diseases of a wide range of hosts (1). Currently, seven X. fastidiosa genomes have been completely sequenced, including citrus-variegated chlorosis strain 9a5c, Pierce’s disease strains Temecula 1 and GB514, almond leaf scorch strains M12 and M23, oleander strain Ann1, and mulberry strain MUL0034.

In 2013, the first confirmed outbreak of X. fastidiosa in the European Union was reported from southern Italy (Apulia), the infection occurring on olive trees affected by olive quick decline syndrome (OQDS) and characterized by extensive scorching and desiccation of leaves and twigs (2). Investigations were therefore initiated to determine the taxonomic allocation, host range, and vector(s) of the bacterial strain associated with OQDS, which was denoted CoDiRO, the abbreviation of the Italian name of the disease.

Genomic DNA was recovered from a pure X. fastidiosa CoDiRO culture from infected periwinkle. A paired-end DNA library was constructed and sequenced using Illumina technology, which resulted in 9,008,814 reads and 345× coverage.

Reads were assembled *de novo* using EDENA, Velvet, and SOAPdenovo (3–5) with different *k*-mers. The best contig assemblies from each program were merged using CISA (6) and scaffolded with SSPACE (7) on the Orione instance of Galaxy (8). This reconstruction resulted in a final assembly of 12 scaffolds with sizes ranging from 1,790 to 678,618 bp and an average scaffold size of 211,911 bp. Scaffolds were ordered on the backbone of the reference genome sequence of X. fastidiosa subsp. *pauca* 9a5c, which, according to multilocus sequence typing, was the most related strain. Where necessary, PCR and Sanger sequencing were performed by primer walking to fix the position of contigs showing conflicting ordering information.

The draft genome of X. fastidiosa CoDiRO consisted of a total of 2,507,614 bp (with a GC content of 51.8%), likely representing >95% of the full genomic sequence since other X. fastidiosa strains have genomes ranging from 2.39 to 2.73 Mbp (9).

The draft genome was annotated through submission to the NCBI Prokaryotic Genome Automatic Annotation Pipeline (PGAAP), resulting in the identification of 6 rRNA genes, 49 tRNA loci, 2,053 protein-encoding genes, and 2 noncoding RNAs. A plasmid of 35,318 bp was also found sharing 98% similarity in the *tra* and *trb* loci with the conjugative plasmid pXF-RIV5 (10), but differing from it for the accessory module containing genes of a toxin-antitoxin system.

Comparative analyses showed that (i) variations (insertion/deletion of nucleotide) exist in genes encoding important virulence factors (*rpf* cluster gene, polygalacturonase-*pglA* gene) that are likely to be involved with recognition of specific host factors, which may influence host specificity, infectivity, and/or development of virulence capacity; and (ii) CoDiRO is genetically related with X. fastidiosa subsp. *pauca* isolates with the highest similarity with an isolate from Central America but not with the X. fastidiosa strain infecting olives in California (11).

### Nucleotide sequence accession numbers.

This whole-genome shotgun project has been deposited at DDBJ/EMBL/GenBank under the accession number JUJW00000000. The version described in this paper is version JUJW01000000.
